# Response to dabrafenib plus trametinib on a rare BRAF mutation (V600_W604 deletion–insertion R) in an advanced non‐small cell lung cancer patient

**DOI:** 10.1111/1759-7714.15330

**Published:** 2024-05-20

**Authors:** Akiko Tamura, Ryoko Inaba Higashiyama, Tatsuya Yoshida, Yaya Satozono, Yuichiro Ohe

**Affiliations:** ^1^ Department of Thoracic Oncology National Cancer Center Hospital Tokyo Japan

**Keywords:** BRAF mutation, BRAF V600_W604 deletion–insertion R, comprehensive genomic profiling, dabrafenib plus trametinib, lung adenocarcinoma

## Abstract

Although dabrafenib plus trametinib has been approved for BRAF V600E mutation positive advanced non‐small cell lung cancer (NSCLC), data on its efficacy against uncommon BRAF mutations are still limited due to their rare frequency. We report a case of 70‐year‐old woman with BRAF V600_W604 deletion–insertion R‐positive stage IVA lung adenocarcinoma, who was successfully treated with dabrafenib plus trametinib. Herein, we discuss the oncogenic role of uncommon BRAF mutations and highlight the importance of performing comprehensive genomic profiling on patients without any targetable gene alterations in companion diagnostics.

## Graphical abstract

A 70‐year‐old woman with stage IVA lung adenocarcinoma underwent comprehensive genomic profiling (CGP) after failure of second‐line therapy, which revealed BRAF V600_W604 deletion–insertion. She was successfully treated with dabrafenib 300 mg/day plus trametinib 2 mg/day. CGP should be considered when initial companion diagnostics do not detect druggable oncogene alterations.

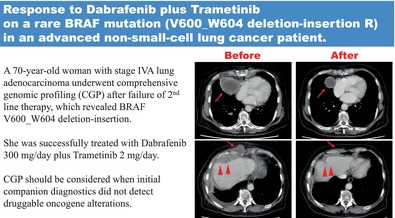

## INTRODUCTION

Dabrafenib plus trametinib has been approved for the treatment of BRAF V600E mutation‐positive advanced non‐small cell lung cancer (NSCLC). Data on its efficacy against other uncommon BRAF mutations are limited because of the rare frequency of BRAF mutations in patients with NSCLC.^1^, ^2^ Herein, we report a case of BRAF V600_W604 deletion–insertion R‐positive stage IVA lung adenocarcinoma that was successfully treated with dabrafenib and trametinib, and the importance of comprehensive genomic profiling tests, even when initial companion diagnostics did not detect druggable oncogene alterations, are discussed.

## CASE REPORT

**FIGURE 1:**
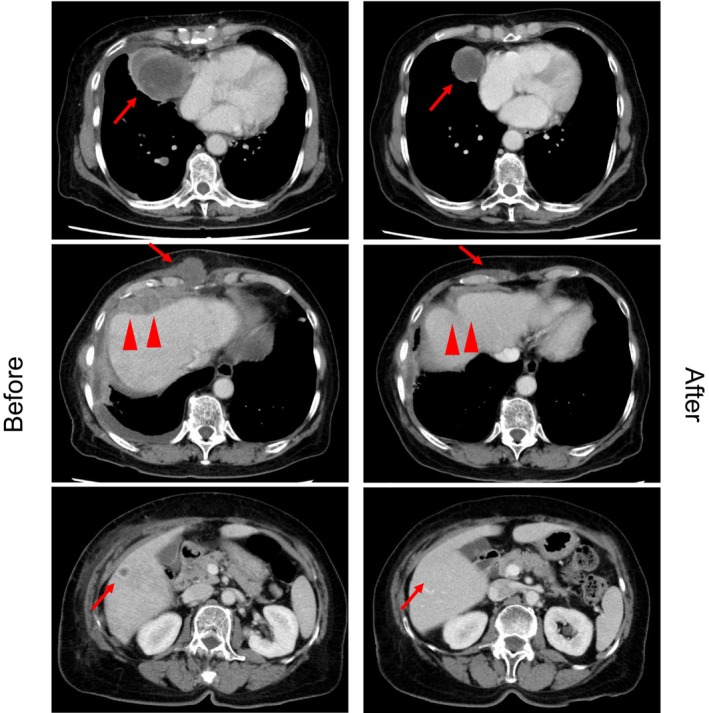
Chest computed tomography at the time of disease progression after 10 cycles of pemetrexed and bevacizumab maintenance therapy (left), and 48 days after dabrafenib (300 mg/day) plus trametinib (2 mg/day) therapy (right).

**FIGURE 2:**
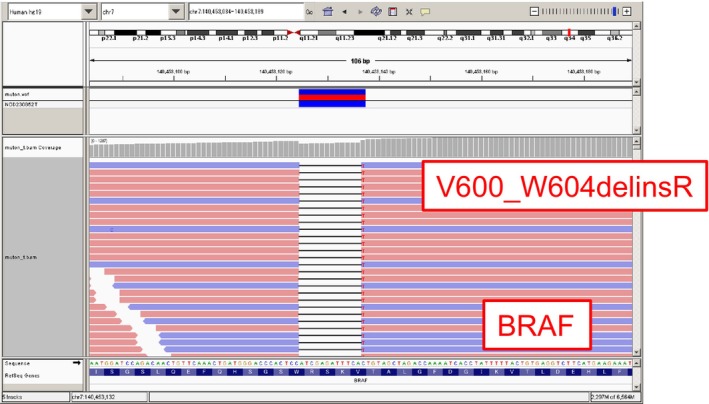
The result of OncoGuide NCC Oncopanel System, revealing V600_W604 deletion–insertion R.

A 70‐year‐old female without a history of smoking with stage IVA (cT4N2M1a) lung adenocarcinoma received pembrolizumab monotherapy as first‐line treatment, given that companion diagnostics (CDx) using amplicon‐based targeted next‐generation sequencing (NGS) (Oncomine Dx Target Test multi‐CDx system, Thermo Fisher Scientific) revealed that she had no driver gene mutation, whereas the PD‐L1 expression TPS (22C3) was 95%. Disease progression was observed in the pleura, intercostal muscles, and mediastinal lymph nodes after 17 cycles. Subsequently, the patient received four cycles of carboplatin, pemetrexed, and bevacizumab, followed by pemetrexed and bevacizumab maintenance therapy. After 10 cycles, the computed tomography (CT) scan revealed new metastases in the bilateral lungs, liver, and skin, in addition to worsening of the existing metastasis (Figure 1, left). Tissue samples were obtained from the pleural dissemination for comprehensive genomic profiling (CGP) using a hybridization capture‐based NGS assay (OncoGuide NCC Oncopanel System, Sysmex Corporation), which revealed BRAF V600_W604 deletion–insertion R^3^ (Figure 2). Thus, the patient commenced dabrafenib 300 mg/day plus trametinib 2 mg/day. On day 6, shrinkage of the cardiophrenic lymph node metastasis and decrease of pleural effusions were observed on chest x‐rays. On day 48, CT tomography confirmed a partial response (Figure 1, right). Unfortunately, the disease progressed in 5.5 months and the patient was moved to palliative care.

## DISCUSSION

BRAF mutations account for 1%–5% of NSCLC.^2^, ^4^ They are divided into three classes depending on their kinase activity and structure: class I is V600 mutations where kinase‐activated BRAF exists as monomers; class II is non‐V600 mutations where kinase‐activated BRAF exists as dimers; and class III is non‐V600 mutations where, although kinase‐impaired, BRAF passes on RAS signals to downstream MEK and ERK by forming heterodimers with CRAF.^5^, ^6^ The most frequent mutation is V600E in class I, which occurs in 35%–50% of all BRAF mutations in NSCLC, and the efficacy of dabrafenib plus trametinib has been shown clinically in advanced NSCLC patients.^1^, ^2^, ^7^ V600K and V600R are also BRAF‐activating class I mutations.^8^

Regarding the molecular structure of BRAF in more detail, over 80% of known BRAF mutations occur in the activation loop (A‐loop) at residues of 594–601 or a phosphate‐binding loop (P‐loop) at residues 464–469 of the kinase domain. In normal circumstances, the interaction between the A‐loop and P‐loop (especially between V600 and F468) stabilizes the inactive conformation of BRAF. However, when BRAF mutation occurred at the A‐loop or P‐loop, these mutations often disrupt the interaction, leading to oncogenic activation of BRAF.^1^, ^9^, ^10^

Our patient revealed V600_W604 deletion–insertion R, which was unclassifiable to any of the three classes described above. Furthermore, although this mutation had been reported in one NSCLC patient, one papillary thyroid carcinoma patient, and one melanoma patient, no data existed on whether this mutation contributed to BRAF activation or showed response to dabrafenib plus trametinib.^1^, ^11^, ^12^ However, our patient's response to therapy suggested it to be a BRAF‐activating mutation, and we suspect that this was due to the alteration of amino acid sequence at the A‐loop leading to disruption of A‐loop and P‐loop interaction, resulting in oncogenic activation of BRAF.

In conclusion, our study described a case of BRAF V600_W604 deletion–insertion R‐positive stage IVA NSCLC that was successfully treated with dabrafenib plus trametinib. Additionally, CGP should be considered when CDx does not reveal any targetable gene alterations, because CGP by hybrid capture‐based NGS can be more efficient in detecting rare variant mutations than amplicon‐based CDx, and thus provides the possibility of applying existing therapies to rare‐variant mutations.

## AUTHOR CONTRIBUTIONS

Akiko Tamura: Writing–original draft preparation. Ryoko Inaba Higashiyama: Data curation. Tatsuya Yoshida: Conceptualization, writing–review and editing. Yaya Satozono: Writing–review and editing. Yuichiro Ohe: Project administration.

## CONFLICT OF INTEREST STATEMENT

Tatsuya Yoshida reports grants from AMGEN, Astrazeneca, Bristol, Medpace, Merck, Daiichi Sankyo, Ono, MSD, Novartis, Chugai, Abbvie, honoraria for lectures from MSD, Astrazeneca, ONO, and Chugai, outside the submitted work.

Yuichiro Ohe reports grants from AstraZeneca, Chugai, Lilly, ONO, BMS, Kyorin, Dainippon‐ Sumitomo, Pfizer, Taiho, Novartis, Takeda, Kissei, Daiichi‐Sankyo, Janssen, consulting fees from AstraZeneca, Chugai, ONO, BMS, Kyorin, Celltrion, PharmaMar, AnHeart Therapeutics Inc., Amgen, Nippon Kayaku, Boehringer Ingelheim, and honoraria for lectures from MSD, AstraZeneca, Chugai, ONO, BMS, Kyorin, Celltrion, PharmaMar, AnHeart Therapeutics Inc., Amgen, Nippon Kayaku, Boehringer Ingelheim, Lilly, Pfizer, Taiho, Daiichi‐Sankyo, Bayer, Kyowa Hakko Kirin, and Eisai, outside the submitted work.

The remaining authors state that they have no conflict of interest.
